# Supplementary material for: Contents of the Sexual and Reproductive Health Subject in the Undergraduate Nursing Curricula of Spanish Universities: A Cross-Sectional Study
Source: Int J Environ Res Public Health. 2021 Oct 31;18(21):11472. doi: 10.3390/ijerph182111472 (PMC8583184; doi:10.3390/ijerph182111472)
Supplement: Supplementary file 1 [file ijerph-18-11472-s001.zip › ijerph-1384946-supplementary.pdf]

Supplementary Table S1. Subtopics of the area of Reproductive Health. (N = 77)

	**Total Universities (N = 77)**		**Public Universities (n = 52)**		**Private Universities (n = 25)**		
	**n**	**%**	**n**	**%**	**n**	**%**	**p**
**I. Human reproduction**							
1. Anatomy of the female reproductive organs	**60**	**77,9**	40	76,9	20	80,0	
2. Physiology of the female reproductive system	**61**	**79,2**	42	80,8	19	76,0	
3. Anatomy and physiology of the male reproductive system 14.2	32	41,6	24	46,2	8	32,0	< 0,05
4. Gametogenesis 10.5	33	42,9	24	46,2	9	36,0	< 0,05
**II. Human prenatal development**							
1. Fertilization, implantation, germ disc	50	64,9	35	67,3	15	60,0	
2. Embryonic period	43	55,8	29	55,8	14	56,0	
3. Fetal period	42	54,5	30	57,7	12	48,0	
4. Development and structure of the placenta 12.5	25	32,5	19	36,5	6	24,0	< 0,05
**III. Pregnancy**							
1. Preconception consultation	34	44,2	26	50,0	8	32,0	< 0,05
2. Pregnancy	**65**	**84,4**	45	86,5	20	80,0	
3. Diagnosis of pregnancy	41	53,2	30	57,7	11	44,0	< 0,05
4. Prenatal care	46	59,7	31	59,6	15	60,0	
5. Prenatal diagnosis	27	35,1	22	42,3	5	20,0	< 0,01
6. Assessment of fetal well-being	31	40,3	25	48,1	6	24,0	< 0,01
7. Health education for pregnant women	33	42,9	28	53,8	5	20,0	< 0,001
8. Nutrition of the pregnant woman	24	31,2	21	40,4	3	12,0	< 0,01
9. Factors that affect the development of pregnancy	24	31,2	20	38,5	4	16,0	< 0,01
10. Drugs in pregnancy	17	22,1	15	28,8	2	8,0	< 0,01
11. Psychosocial aspects in pregnancy	26	33,8	23	44,2	3	12,0	< 0,001
12. Maternity education programs	25	32,5	22	42,3	3	12,0	< 0,001
13. Assisted reproduction	20	26,0	18	34.6	2	8.0	< 0,01

	Total Universities (N = 77)		Public Universities (n = 52)		Private Universities (n = 25)		
	n	%	n	%	n	%	p
**IV. Health problems during pregnancy**							
1. Bleeding problems	**63**	**81,8**	44	84,6	19	76,0	
2. Hypertensive states	**64**	**83,1**	45	86,5	19	76,0	< 0,05
3. Diabetes	56	72,7	40	76,9	16	64,0	< 0,05
4. Anemias	44	57,1	30	57,7	14	56,0	
5. Heart problems. Heart disease	43	55,8	30	57,7	13	52,0	
6. Varicose syndromes	39	50,6	27	51,9	12	48,0	
7. Endocrine problems	46	59,7	31	59,6	15	60,0	
8. Renal problems	43	55,8	29	55,8	14	56,0	
9. Hyperemesis	51	66,2	34	65,4	17	68,0	
10. Oral and digestive problems	44	57,1	29	55,8	15	60,0	
11. Respiratory problems	43	55,8	29	55,8	14	56,0	
12. Neurological problems	42	54,5	29	55,8	13	52,0	
13. Dermatological problems	40	51,9	27	51,9	13	52,0	
14. Neoplastic problems	44	57,1	31	59,6	13	52,0	
15. Infectious problems	**60**	**77,9**	40	76,9	20	80,0	
16. AIDS	48	62,3	33	63,5	15	60,0	
17. Consumption of toxic substances	43	55,8	29	55,8	14	56,0	
18. Perinatal hemolytic disease	42	54,5	29	55,8	13	52,0	
19. Alterations of the placenta, membranes, cord and amniotic fluid	49	63,6	31	59,6	18	72,0	< 0,05
20. Multiple gestation	51	66,2	37	71,2	14	56,0	< 0,05
21. Threat of premature labor	50	64,9	35	67,3	15	60,0	
22. Prolonged pregnancy	44	57,1	30	57,7	14	56,0	
23. Intrauterine growth retardation	46	59,7	32	61,5	14	56,0	
24. Psychological aspects of pregnancy at risk	42	54,5	28	53,8	14	56,0	
25. Mental health disorders	40	51,9	27	51,9	13	52,0	
26. Antepartum fetal death	41	53,2	29	55,8	12	48,0	

	Total Universities (N = 77)		Public Universities (n = 52)		Private Universities (n = 25)		
	**n**	**%**	**n**	**%**	**n**	**%**	**p**
27. Drugs in health problems	39	50,6	27	51,9	12	48,0	
28. Genetic counseling and prenatal diagnosis. Reproductive advice	42	54,5	29	55,8	13	52,0	
**V. Delivery**							
1. Labor and delivery	**61**	**79,2**	39	75,0	22	88,0	< 0,05
2. Factors involved in childbirth	36	46,8	22	42,3	14	56,0	< 0,05
3. Start of labor and delivery periods	50	64,9	32	61,5	18	72,0	< 0,05
4. Mechanism of delivery in vertex presentation	29	37,7	20	38,5	9	36,0	
5. Assistance and care of the woman in childbirth	48	62,3	31	59,6	17	68,0	
6. Assessment of fetal well-being	31	40,3	22	42,3	9	36,0	
7. Pain relief in labor	37	48,1	23	44,2	14	56,0	< 0,05
8. Attention to normal delivery	51	66,2	35	67,3	16	64,0	
9. Drugs in childbirth	22	28,6	15	28,8	7	28,0	
10. Obstetric analgesia and anesthesia	24	31,2	16	30,8	8	32,0	
11. Assistance and care of the woman in directed childbirth	30	39,0	21	40,4	9	36,0	
12. Psychological aspects of women during labor	25	32,5	17	32,7	8	32,0	
13. Different alternatives in obstetric care	24	31,2	16	30,8	8	32,0	
**VI. Complications in labor and delivery**							
1. Dystocic delivery	54	70,1	33	63,5	21	84,0	< 0,01
2. Alterations in fetal statics	35	45,5	24	46,2	11	44,0	
3. Obstetric instrumentation and interventions	39	50,6	27	51,9	12	48,0	
4. Birth trauma	34	44,2	24	46,2	10	40,0	
5. Premature rupture of membranes	42	54,5	29	55,8	13	52,0	
6. Intrapartum fever	31	40,3	22	42,3	9	36,0	
7. Risk of loss of fetal well-being	33	42,9	24	46,2	9	36,0	< 0,05
8. Preterm delivery	35	45,5	25	48,1	10	40,0	
9. Birth abnormalities	35	45,5	24	46,2	11	44,0	

	Total Universities (N = 77)		Public Universities (n = 52)		Private Universities (n = 25)		
	n	%	n	%	n	%	p
10. Childbirth and puerperium hemorrhages	34	44,2	22	42,3	12	48,0	
11. Drugs in Complicated Labor	31	40,3	21	40,4	10	40,0	
12. Special obstetric emergency situations	39	50,6	28	53,8	11	44,0	
**VII. Puerperium and lactation**							
1. The puerperium	**65**	**84,4**	45	86,5	20	80,0	
2. Drugs in the puerperium and lactation	14	18,2	12	23,1	2	8,0	< 0,05
3. Psychosocial adaptation of the puerperal woman	21	27,3	19	36,5	2	8,0	< 0,01
4. Lactation	22	28,6	18	34,6	4	16,0	< 0,05
5. Breastfeeding	55	71,4	36	69,2	19	76,0	
6. Home care in the puerperium	21	27,3	18	34,6	3	12,0	< 0,01
7. Postpartum programs	16	20,8	14	26,9	2	8,0	< 0,05
**VIII. Health problems in the puerperium**							
1. Puerperal fever	41	53,2	29	55,8	12	48,0	
2. Thromboembolic disease	49	63,6	35	67,3	14	56,0	< 0,05
3. Pelvic joint injuries. Scar dehiscences	41	53,2	29	55,8	12	48,0	
4. Psychological disorders in the puerperium	44	57,1	32	61,5	12	48,0	< 0,05
5. The grieving process in motherhood and reproduction	42	54,5	30	57,7	12	48,0	
6. Drugs in the puerperium with complications	41	53,2	30	57,7	11	44,0	< 0,05
**IX. Women's reproductive health care**							
1. Women's Sexual and Reproductive Health Care Programs	20	26,0	15	28,8	5	20,0	
2. Attention to women's sexual and reproductive health	37	48,1	24	46,2	13	52,0	
3. Family planning and contraceptive counseling	38	49,4	32	61,5	6	24,0	< 0,001
4. Natural methods	28	36,4	22	42,3	6	24,0	< 0,05
5. Barrier methods	28	36,4	22	42,3	6	24,0	< 0,05
6. Hormonal contraception	21	27,3	19	36,5	2	8,0	< 0,01

	Total Universities (N = 77)		Public Universities (n = 52)		Private Universities (n = 25)		
	n	%	n	%	n	%	p
7. Intrauterine device	19	24,7	16	30,8	3	12,0	< 0,05
8. Surgical methods	16	20,8	12	23,1	4	16,0	
9. Contraception in special situations	12	15,6	11	21,2	1	4,0	< 0,05
10. Voluntary termination of pregnancy	12	15,6	11	21,2	1	4,0	< 0,05
11. Puberty and adolescence	29	37,7	18	34,6	11	44,0	
12. Climacteric and menopause	**58**	**75,3**	38	73,1	20	80,0	
13. Drugs in sexual and reproductive health care	8	10,4	8	15,4	0	0,0	< 0,05
**X. Care for women with reproductive health problems**							
1. Alterations of the menstrual cycle	45	58,4	31	59,6	14	56,0	
2. Sexually transmitted infections	51	66,2	34	65,4	17	68,0	
3. Infectious processes of the reproductive system	37	48,1	24	46,2	13	52,0	
4. Pelvic pain	17	22,1	12	23,1	5	20,0	
5. Infertility and sterility	38	49,4	25	48,1	13	52,0	
6. Malformations of the genital tract	18	23,4	12	23,1	6	24,0	
7. Benign alterations of the female genital tract	32	41,6	23	44,2	9	36,0	
8. Changes in genital statics	15	19,5	9	17,3	6	24,0	
9. Urinary incontinence	15	19,5	12	23,1	3	12,0	< 0,05
10. Benign breast disorders	33	42,9	19	36,5	14	56,0	< 0,05
11. Early diagnosis of breast and gynecological cancer	36	46,8	24	46,2	12	48,0	
12. Gynecological and breast cancer	47	61,0	30	57,7	17	68,0	< 0,05
13. Drugs in sexual and reproductive health problems	8	10,4	7	13,5	1	4,0	
14. Gynecological Surgical Interventions	12	15,6	8	15,4	4	16,0	
15. The hospitalized gynecological patient	12	15,6	8	15,4	4	16,0	
**XI. Epidemiology and demography in reproductive health**							
1. Epidemiology	7	9,1	5	9,6	2	8,0	
2. Diagnostic tests in reproductive health processes	9	11,7	6	11,5	3	12,0	

	Total Universities (N = 77)		Public Universities (n = 52)		Private Universities (n = 25)		
	n	%	n	%	n	%	p
3. Maternal and reproductive health demographics	3	3,9	2	3,8	1	4,0	
4. Reproductive morbidity	2	2,6	1	1,9	1	4,0	
**XII. Socio-anthropological aspects in reproductive health**							
1. Sociocultural influences on reproductive health care	4	5,2	2	3,8	2	8,0	
2. Gender perspective	10	13,0	8	15,4	2	8,0	
3. The family and family forms	6	7,8	4	7,7	2	8,0	
4. Anthropology of motherhood	3	3,9	2	3,8	1	4,0	
5. Women and immigration	1	1,3	0	0,0	1	4,0	
**XIII. Women's Health Education and Research**							
1. Education for women's health	30	39,0	22	42,3	8	32,0	< 0,05
2. Individual health education and group health education	6	7,8	3	5,8	3	12,0	
3. Learning in adults	5	6,5	3	5,8	2	8,0	
4. Health attitudes and behaviors	10	13,0	7	13,5	3	12,0	
5. The communicative process in education for women's health	8	10,4	4	7,7	4	16,0	
6. Teaching strategies	11	14,3	6	11,5	5	20,0	
7. Application of group dynamics in educational programs for women's health	1	1,3	1	1,9	2	8,0	
8. Gender perspective in health education programs	12	15,6	7	13,5	5	20,0	
9. Development of a health education program for women	2	2,6	0	0,0	2	8,0	
10. Research in Women's Health	1	1,3	1	1,9	0	0,0	
**XIV. Legislation and Ethics**							
1. Rights of women and the newborn	3	3,9	3	5,8	0	0,0	
2. Labor rights and maternity	3	3,9	3	5,8	0	0,0	
3. Ethical aspects in women's health care	3	3,9	3	5,8	0	0,0	
4. Legal responsibilities	3	3,9	3	5,8	0	0,0	
5. Control and protection of professional practice	3	3,9	3	5,8	0	0,0	

	Total Universities (N = 77)		Public Universities (n = 52)		Private Universities (n = 25)		
	**n**	**%**	**n**	**%**	**n**	**%**	**p**
6 Legislation and ethics at birth	4	5,2	10	19,2	4	16,0	
7. Legislation and ethics in assisted reproduction	2	2,6	14	26,9	6	24,0	
8. Legislation and ethics in contraception and interruption of pregnancy	4	5,2	10	19,2	4	16,0	
9. Crimes against sexual freedom	5	6,5	4	7,7	1	4,0	
10. Adoption and foster care of a child	3	3,9	3	5,8	0	0,0	
11. Surrogacy	1	1,3	1	1,9	0	0,0	
**XV. Women's Health Services Administration**							
1. Health systems models	2	2,6	2	3,8	0	0,0	
2. Analysis of the situation	2	2,6	2	3,8	0	0,0	
3. The Maternal-Infant Hospital	2	2,6	2	3,8	0	0,0	
4. Primary-Community Care	3	3,9	2	3,8	1	4,0	
5. Development of objectives in Women's Health	2	2,6	2	3,8	0	0,0	
6. Registration systems in Women's Health	2	2,6	2	3,8	0	0,0	
7. Coordination between the different levels of healthcare	2	2,6	2	3,8	0	0,0	
8. Development of healthcare protocols	2	2,6	2	3,8	0	0,0	
9. Process and program evaluation	3	3,9	2	3,8	1	4,0	

Bold: Percentage ≥ 75%
